# Linking Genomo- and Pathotype: Exploiting the Zebrafish Embryo Model to Investigate the Divergent Virulence Potential among *Cronobacter* spp.

**DOI:** 10.1371/journal.pone.0158428

**Published:** 2016-06-29

**Authors:** Athmanya K. Eshwar, Ben D. Tall, Jayanthi Gangiredla, Gopal R. Gopinath, Isha R. Patel, Stephan C. F. Neuhauss, Roger Stephan, Angelika Lehner

**Affiliations:** 1 Institute for Food Safety and Hygiene, University of Zurich, Zurich, Switzerland; 2 Center for Food Safety and Applied Nutrition, Food and Drug Administration, Laurel, Maryland, United States of America; 3 Institute of Molecular Life Sciences, University of Zurich, Zurich, Switzerland; University Medical Center Utrecht, NETHERLANDS

## Abstract

Bacteria belonging to the genus *Cronobacter* have been recognized as causative agents of life-threatening systemic infections primarily in premature, low-birth weight and immune-compromised neonates. Apparently not all *Cronobacter* species are linked to infantile infections and it has been proposed that virulence varies among strains. Whole genome comparisons and *in silico* analysis have proven to be powerful tools in elucidating potential virulence determinants, the presence/absence of which may explain the differential virulence behaviour of strains. However, validation of these factors has in the past been hampered by the availability of a suitable neonatal animal model. In the present study we have used zebrafish embryos to model *Cronobacter* infections *in vivo* using wild type and genetically engineered strains. Our experiments confirmed the role of the RepF1B-like plasmids as “virulence plasmids” in *Cronobacter* and underpinned the importantce of two putative virulence factors—*cpa* and *zpx*—in *in vivo* pathogenesis. We propose that by using this model *in vivo* infection studies are now possible on a large scale level which will boost the understanding on the virulence strategies employed by these pathogens.

## Introduction

*Cronobacter* (C.) spp. are Gram-negative, rod-shaped, non- sporeforming, motile bacteria of the family *Enterobacteriaceae*. The genus *Cronobacter*–as proposed in 2008 –currently consists of seven species according to the “List of prokaryotic names with standing in nomenclature” (http://www.bacterio.net/allnamesac.html, viewed, June 6th 2016) and encompasses organisms that have previously been identified as *Enterobacter sakazakii* [1, 2]. The extension of the genus *Cronobacter* by three non-pathogenic *Enterobacter* species (*E*. *pulveris*, *E*. *helveticus* and *E*. *turicensis*), as proposed by Brady et al. [3] was withdrawn as the genus membership was experimentally disproved in the study by Stephan et al. [4].

*Cronobacter* spp. are regarded as opportunistic pathogens linked with life-threatening infections, particularly in premature (< 37 weeks of gestation stage), low-birth weight (< 2500 g) or immuno-compromised neonates and infants less than 4 weeks of age [5, 6] and its occurrence has been epidemiologically linked to the consumption of reconstituted intrinsically or extrinsically contaminated powdered infant formula (PIF) [7, 8].

Clinical symptoms include necrotising enterocolitis (NEC), bacteremia and meningitis, with case fatality rates ranging between 40 and 80% being reported [9, 10].

Apparently not all *Cronobacter* species are linked to infantile infections and it has been proposed that virulence varies among strains. Thus *C*. *sakazakii*, *C*. *malonaticus*, and *C*. *turicensis* are the primary pathogenic species which cause the majority of severe illnesses neonates [11]. Some studies suggest that *C*. *malonaticus* may be more of an adult pathogen than *C*. *sakazakii* or *C*. *turicensis* are [12]. Other species of *Cronobacter* include *C*. *universalis*, *C*. *condimenti*, *C*. *muytjensii*, and *C*. *dublinensis*. Except for *C*. *condimenti*, all other *Cronobacter* species cause a variety of infections in humans [1, 7].

Applying a multilocus sequence typing scheme (MLST) to *C*. *sakazakii* strains revealed distinct pathovars which are clonal lineages of particular clinical significance, namely clonal comlpex 4 (CC4) that contains multilocus sequence type 4 (ST4), as well as ST12. These are strongly associated with invasive meningitis and NEC cases. In addition, *C*. *malonaticus* clonal complex 7 was found to be linked with adult infections [7, 13, 14, 15].

Whole genome sequencing efforts and *in silico* analysis revealed a substantial amount of genotypic variation and identified potential determinants encoding for potential virulence determinants, present in some strains but absent in others which may explain that not all *Cronobacter* spp. are equally virulent and cause invasive disease such as bacteremia and meningitis [11, 16, 17]. However, the validation of putative virulence components has been hampered by the availability of suitable neonatal animal models. So far, *in vivo* studies have concentrated on the neonatal rat, mouse or gerbil as model organisms [17,18,19,20]. Although valuable information has been obtained from these studies, the lack of possibilities for a real-time analysis and the need for laborious and invasive sample analysis limits the use of mammalian experimental animals.

Alternatively, the nematode *Caenorhabditis elegans* has been used to study *Cronobacter* virulence factors and host immune responses [21]. Thus, the role of the *Cronobacter sakazakii* lipopolysaccharide (LPS) and the p38 MAPK pathway as a major factor in host immune response against LPS-mediated challenges has been elucidated using this model [22]. However, invertebrates are genetically not closely related to humans and their immune system shows many differences from the human immune system.

Zebrafish (*Danio rerio*) are increasingly used as model to study infections with human pathogens as they offer distinct advantages over mammalian models, such as mice and rats or invertebrate animal models such as the nematode *C*. *elegans*. Being vertebrates, zebrafish are evolutionarily closer to humans than are nematodes and they are easier to work with and to study than mice, but retain the advantage of a similar immune system [23]. The execution of large-scale infection studies in zebrafish is possible due to their fecundity and small size. In a recent study we adapted the zebrafish embryo model to study *Cronobacter* infections [24]. This model proved especially useful, since in embryos only the innate immune system is displayed which resembles the situation during infection in premature infants and neonates.

In the current study we employed the recently developed zebrafish embryo model to (1) determine the virulence spectrum displayed among species and strains within the *Cronobacter* genus, (2) define the role of the proposed “virulence plasmids”in *Cronobacter* spp. and (3) confirm the influence of two putative virulence determinants—the plasmid encoded Cpa and the chromosomally encoded Zpx—in *in vivo* pathogenesis.

## Material and Methods

### Bacterial strains and culture conditions

The description of the wild type strains used in the study is given in Table 1. The two plasmid-cured strains *C*. *turicensis* LMG 23827^T^ΔpCTU1 and *C*. *sakazakii* ATCC BAA-894 ΔpESA3 were constructed in the study by Franco et al. [25]. Those strains together with the *C*. *sakazakii* strain E899, which is naturally devoid for the pESA3 plasmid were provided by CFSAN, FDA, USA. *Cronobacter* spp. and *E*. *coli* strains were grown at 37°C in Luria-Bertani (LB) broth with shaking (210 rpm) or on LB agar. Antibiotics were added when required at the following concentrations: ampicillin (100 mg L^-1^), chloramphenicol (30 mg L^-1^ and nalidixic acid (256 mg L^-1^).

**Table 1:** Wild type strains used in the zebrafish embryo infection experiments.

Species	Strain ID ATCC^a^ LMG^b^, other*	O type^c^	Sequence type^d^	Source and/or reference
*Cronobacter condimenti*	LMG 26250^T^	O1	98	Spiced meat [2]
*Cronobacter dublinensis subsp*. *dublinensis*	LMG 23823^T^	O1b	106	Milk powder processing environment [1]
*Cronobacter dublinensis subsp*. *lactaridi*	LMG 23815	O1a	79	Milk powder processing environment [1]
*Cronobacter dublinensis subsp*. *lausannensis*	LMG 23824	O2	80	Basin of a water fountain [1]
*Cronobacter malonaticus*	LMG 23826^T^	O2	7	Human breast abscess [1]
*Cronobacter muytjensii*	ATCC 51329^T^	O2	81	Unknown [1]
*Cronobacter sakazakii*	ATCC 29544^T^	O1	8	Child throat [1]
*Cronobacter sakazakii*	ATCC BAA-894	O1	1	Milk powder [26]
*Cronobacter sakazakii*	E899*	O2	4	Clinical
*Cronobacter turicensis*	LMG 23827^T^	O1	19	Neonate [1]
*Cronobacter universalis*	LMG 26249^T^	O1	54	Water [1, 2]
*Escherichia coli*	Xl1 blue	n. a.	n.a.	Agilent

^a^: ATCC = American Type Culture Collection, Manassas, USA

^b^: LMG = BCCM/LMG Laboratorium voor Microbiologie, Universiteit Gent, Gent, Belgium

*: Type culture collection Institute for Food Safety and Hygiene, University Zurich, Zurich, Switzerland, O serotyped and MLST typed by Center for Food Safety and Applied Nutrition FDA, Laurel, Maryland, USA

^c, d^: O (Sero)type and MLST Sequence type designations retrieved from Ogrodzki and Forsythe [27].

n. a.: not applicable

A nalidixic acid resistant strain of *C*. *sakazakii* ATCC BAA-894(Nal256) was constructed according to the method by Johnson et al. [28]. Strain *C*. *sakazakii* ATCC BAA-894Δ*cpa* was created during transconjugation experiments and was consequently nalidixic acid resistant as well. Both strains were used during quantitative zebrafish embryo infection experiments. Cultures for experiments were grown in LB supplemented with nalidixic acid (256 mg L^-1^)

For microinjection experiments, the bacteria were grown to stationary phase in LB overnight (approx. 12 hours) at 37°C, harvested by centrifugation at 5000 x *g* for 10 min and washed once in 10 ml of Dubelcco`s phosphate buffered saline (DPBS, Life Technologies.) After a second centrifugation step, the cells were resuspended in DPBS, and appropriate dilutions were prepared in DPBS.

### DNA extraction and manipulations

Chromosomal DNA was isolated using the DNeasy Blood and Tissue kit (Qiagen), plasmids were extracted with the QIAprep Spin Miniprep or Plasmid Midi kits (Qiagen) following the manufacturer’s instructions. For purification purposes (PCR, restriction digestion, agarose gel purification) the Qiagen MinElute PCR Cleanup kit or MinElute Gel Purification kit was employed. Enzymes and respective buffers were obtained from Roche Molecular Diagnostics and used according to the manufacturer`s instructions.

### Construction of *C*. *turicensis* LMG 23827^T^Δ*zpx* and complementation

Bacterial strains, plasmids and primers used for the construction of the mutant are listed in Table 2. An isogenic mutant of the *C*. *turicensis* LMG 23827^T^ devoid of the *zpx* (CTU_31020) gene was constructed following the protocol described by Philippe et al. [29]. Primers were designed based on the whole genome sequence of *Cronobacter turicensis* LMG 23827^T^ (RefSeq accession numbers NC_013282 to NC_013285, GenBank accession numbers FN543093 to FN543096). Briefly, two flanking fragments (upstream, downstream) of the *zpx* gene were amplified by PCR using oligonucleotide primers zpxF1f (containing a *XbaI* recognition site), zpxF1r (containing a *XhoI* restriction site), zpxF2fmod (containing a *XhoI* recognition site), zpxF2r (containing a *XbaI* recognition site). The amplification mixes contained 0.4 μM of primers, 1 x AccuPrime (Invitrogen) buffer 2 (60 mM Tris-SO_4_ (pH 8.9), 18 mM (NH_4_)_2_SO_4_, 2 mM MgSO_4_, 2 mM dGTP, 0.2 mM dATP, 0.2 mM dTTP, 0.2 mM dCTP, thermostable AccuPrimeTM protein, 1% glycerol), 4% dimethylsulfoxid (DMSO), 2 U AccuPrime Taq DNA Polymerase High Fidelity (Invitrogen) and 50 ng of template DNA. Following PCR conditions were used for the amplification: 95°C for 120 s followed by 34 cycles of 95°C for 30 s, 68°C for 270 s and a final elongation step at 68°C for 300 s. The resulting fragments were digested with *XbaI* and *XhoI* and ligated into the suicide vector pDS132 digested with *XbaI*. The construct pDS132::Δ*zpx* was transformed into *E*. *coli* SM10 λpir via electroporation. The resulting strain *E*. *coli* SM10 λpir/ pDS132::Δ*zpx* served as donor strain for conjugative transfer of the plasmid into *C*. *turicensis* LMG 23827^T^_Nal^R^. Transconjugants were selected on LB agar plates supplemented with both nalidixic acid 256 mg L^-1^ and chloramphenicol 30 mg L^-1^. The genetic structure of the mutant was confirmed by the presence of two amplification products—one representing the chromosomal wild type *zpx* allele and a second product representing the truncated (Δ*zpx*) allele originating from the (integrated) pDS132::Δ*zpx*—after PCR using primer pair zpxContf, zpxContr employing the above mentioned AccuPrime amplification mixture (without DMSO) and following amplification conditions: 95°C for 120 s followed by 32 cycles of 95°C for 30 s, 52°C for 210 s and a final elongation step at 68°C for 300 s. The resulting amplification products were 1101 bp (wt *zpx* allel) and 240 bp (Δ*zpx* allel).

**Table 2:** Material used for *zpx* mutant construction and complementation.

Strains/plasmids/primers	Genotype/characteristic(s)/sequences	Source or reference
Mutant construction strains		
*C*. *turicensis* LMG 23827^T^_Nal^R^	Acceptor for transconjugation, Nal^R^	[30]
*E*. *coli* SM10 λpir	Host for pDS132::Δ*rpfF*, pDS132::Δ*rpfR* construct generation*; thi*, *thr*, *leu*, *tonA lacY supE recA*::RP4-2-Tc::Mu, Km, *λpir*	[31]
*E*. *coli* DH5α λpir / pDS132	Host for cloning vector pDS132; *sup* E44, Δ*lacU*169 (Φ80*lacZ*ΔM15), *recA1*, *endA1*, *hsdR17*, *thi-1*, *gyrA96*, *relA1*, *λpir*, Cam^R^	[32]
*E*. *coli* SM10 λpir / pDS132::Δ*zpx*	Donor for transconjugation, harbouring construct pDS132::Δ*zpx*, Cam^R^	This study
Plasmids		
pDS132	Low copy cloning vector R6K *ori*, *mobRP4*, *cat*, *sacB*, Cam^R^	[29]
pDS132::Δ*zpx*	Δ*zpx* cloned into pDS132, Cam^R^	This study
Primers		
zpxF1f	5`- AGC TCT AGA AGC GGT CGG AAG AGC CTT TGG—3`	This study
zpxF1r	5`- TAT CTC GAG GCC ATG ATC GAT AAT GCG GCG—3`	This study
zpxF2fmod	5`- GGG CTC GAG GCT CAC TCT CGC AGA ATG CGG—3`	This study
zpxF2r	5`- TGA TCT AGA GGT CTG GTG CTG GTT CAT ACC—3`	This study
zpxConf	5`- CTA TAC TGC AAG TGT TGG—3`	This study
zpxConr	5`- CGT CAT CCG TCA GAT CTG—3`	This study
Complementation		
*C*. *turicensis* LMG 23827^T^	Template for amplification of *zpx* CDS	[33]
*C*. *turicensis* LMG 23827^T^Δ*zpx*	Zpx CDS mutant, cloning host for pQE-30, pQE-30::*zpx*	This study
*C*. *turicensis* LMG 23827^T^Δ*zpx* / pQE-30	Mutant transformant harbouring expression cloning vector pQE-30, Amp^R^	This study
*C*. *turicensis* LMG 23827^T^Δ*zpx* / pQE-30::*zpx*	Mutant transformant harbouring construct pQE-30::*rpfF*, Amp^R^	This study
Plasmid		
pQE-30	Cloning/expression vector, Amp^R^	Qiagen
Primers		
rpfFComplf	5`- AAA GCA TGC AAC CAG TCA CGT TAT CCA ACC- 3`	This study
rpfFComplr	5`- AAA CCC GGG CGT CGG CGT CAT CCG TCA GAT CTG—3`	This study
Insert control		
pQE-30mcsf	5`- CGG ATA ACA ATT TCA CAC AG- 3`	Qiagen
pQE-30r	5`- GTT CTG AGG TCA TTA CTG G- 3`	Qiagen

Outcrossing was performed by plating serial dilutions of confirmed transconjugants onto LB agar plates supplemented with 5% sucrose and no NaCl. Successful allelic exchange was verified in selected chloramphenicol sensitive and and sucrose resistant strains by the presence of the mutant allele after PCR using the above mentioned procedure.

For complementation the *zpx* gene was amplified using primers zpxComplf and zpxComplr (containing an *SphI* and *XmaI* site respectively) and using the above mentioned Accuprime mixture (without DMSO) and following conditions: 95°C for 120 s followed by 32 cycles of 95°C for 30 s, 64°C for 30 s, 68°C for 120 s and a final elongation step at 68°C for 300 s. The resulting fragments were cloned into pQE-30 expression vector and the resulting plasmid pQE-30::*zpx* transformed into *E*. *coli* Xl1blue or *C*. *turicensis* LMG 23827^T^Δ*zpx*.

### Construction of *C*. *sakazakii* ATCC BAA-894Δ*cpa* and complementation

The construction of the *cpa* isogenic mutant, was completed according to the method published by Franco et al. [34]. For complementation experiments, the *cpa* was cloned into pQE-30 expression vector (Qiagen) by PCR amplification using the primers *XmaI*, (5`- AAA CCC GGG AAT AAG AAA CTT ATT GTC GTG GCG- 3`) and *SalI*, (5`- AAA GTC GAC AAC CCG CCG GCA GCG GG- 3`). The recombinant plasmid was transformed into *E*. *coli* Xl1blue or *C*. *sakazakii* ATCC BAA-894Δ*cpa*.

### Zebrafish infection studies

Zebrafish (*Danio rerio*) strains used in this study were albino lines. Husbandry, breeding and microinjection of approx. 50 CFU of bacteria into the yolk sac of 2 dpf embryos was performed following the procedure described in the study by Fehr et al. [24]. A total of thirty embryos (10 x 3) were injected per individual experiment (i.e. per strain).

A set of uninjected embryos, incubated in E3 maintenance medium (5 mM NaCl, 0.17 mM KCl, 0.33 mM CaCl_2_, 0.33 mM MgSO_4_) was included in order to determine the quality of the embryos; embryos injected with DPBS served as controls. Prior to injection, embryos were manually dechorionated and anesthetized with 200 mg L^-1^ buffered tricaine (Sigma-Aldrich).

After injection, the infected embryos were allowed to recover in a petri dish with fresh E3 medium for 15 min. Injected embryos were transferred into 24-well plates (1 embryo per well) in 1 ml E3 medium per well, incubated at 28°C and inspected for survival under a stereomicroscope. At regular time points after infection, the number of dead larvae was determined visually based on the absence of a heartbeat. Experiments were carried out until 96 hours post infection (hpi). Surviving embryos were euthanized with an overdose of 4 g L^-1^ buffered tricaine at the end of the experiments. Generally, with the assessment of discomfort and pain by behavioral observations, animals were euthanized by overdose of tricaine by prolonged immersion and were left in the solution for at least 10 minutes following cessation of opercular movement. Since pain perception has not been developed at these earlier stages (4 dpf– 7 dpf), this is not considered a painful procedure.

The maximum age reached by the embryos during experimentation was 144 hpf. Embryos had not reached free feeding stage then. In addition, research was conducted with approval (NO 216/2012) from the Veterinary Office, Public Health Department, Canton of Zurich (Switzerland) allowing experiments with embryos and larvae older than 120 dpf. The applied methods were carried out following the approved guidelines.

### Survival assay

Thirty 2 dpf embryos were microinjected as mentioned above and maintained individually in 24-well plates in E3 medium at 28°C. Growth behaviour of strains *C*. *sakazakii* ATCC BAA-894 and *C*. *sakazakii* ATCC BAA-894Δ*cpa* was monitored until 72 hpi.

### Bacterial enumeration by plate counting

The larvae were transferred to 1.5 ml centrifuge tubes and disintegrated by repeated pipetting and vortexing for 3 min in 1 mL of DPBS supplemented with 1% Triton X- 100 (Sigma-Aldrich). Subsequently, serial dilutions of this mixture were plated onto LB plates supplemented with 256 mg L^-1^ nalidixic acid. The plates were incubated up to 48 h at 37°C.

### Statistical analysis

Kaplan Meier survival analysis, log rank (Mantel-Cox) test and graphs were performed using GraphPad Prism 6 (GraphPad Software, San Diego, USA). Experiments were executed in triplicates (i.e. 10 embryos x 3 per bacterial strain).

## Results

### Pathogenicity varies within the *Cronobacter* genus and among strains

**Fig 1:**
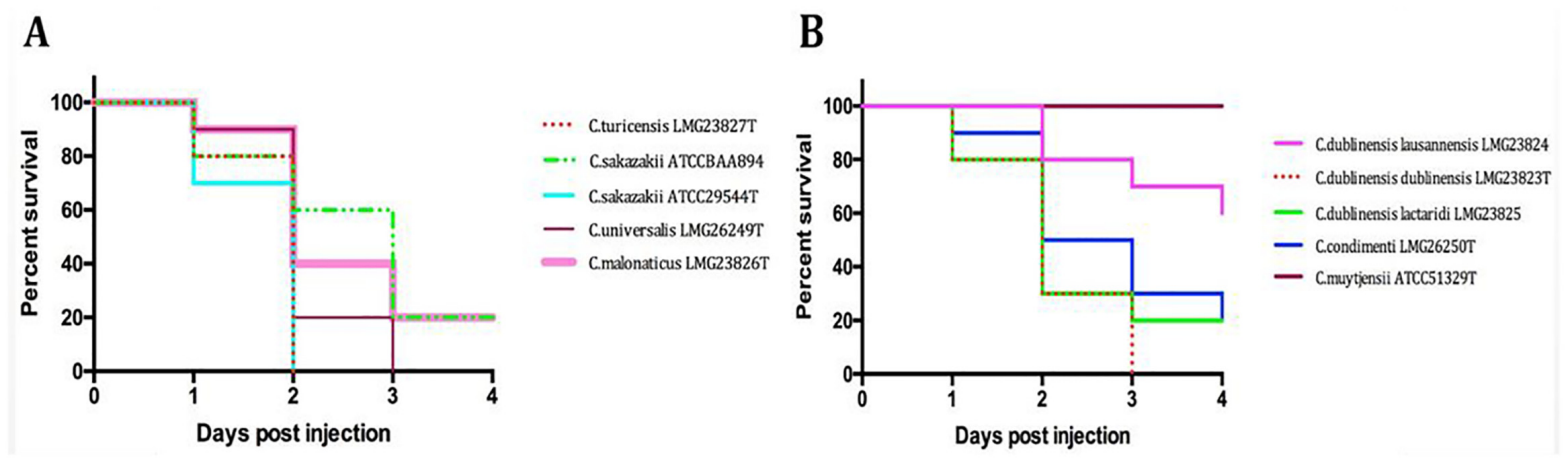
A, B. Zebrafish embryo infection experiments performed with *Cronobacter* wt strains. n = 30 embryos per strain, p = < 0.0001.

Infection experiments using 10 strains—the type strains of the 7 species and 2 subspecies within the *Cronobacter* genus plus one additional *C*. *sakazakii* strain ATCC BAA-894—revealed a substantial degree of variation as determined from the mortality rate in the embryos. Hundred per cent mortality was observed within 36 hours post inoculation (hpi) in infection experiments using *C*. *sakazakii* ATCC 29544^T^ and *C*. *turicensis* LMG 23827^T^ (Fig 1A and 1B). This rate was also reached during experiments using *C*. *universalis* NCT9529^T^ and *C*. *dublinensis* ssp. *dublinensis* LMG 23823^T^ although although death of the embryos occurred at a later time point (72 hpi). For *C*. *sakazakii* ATCC BAA-894 the highest mortality rate (82%) was reached at 96 hpi. Also eighty per cent mortality was observed for *C*. *malonaticus* LMG23826^T^, *C*. *dublinensis* ssp. *lactaridi* LMG 23825 and *C*. *condimenti* LMG 26249^T^ at 96 hpi. For *C*. *dublinensis* ssp. *lausannensis* LMG 23824 infection experiments, a 40% mortality rate was observed at 96 hpi and *C*. *muytjensii* ATCC 51329^T^ was completely avirulent (Fig 1A and 1B).

### The role of pESA3 and pCTU1 as “virulence plasmids” is confirmed *in vivo*

In one of the first comparative genomics studies performed on whole genomes available for the two *Cronobacter* species *C*. *sakazakii* ATCC BAA-894 and *C*. *turicensis* z3032 (= LMG 23827^T^) by Franco et al. [25] it was reported that multiple plasmids were present in the strains including two homologous plasmids identified as pESA3 in *C*. *sakazakii* BAA-894 and pCTU1 in *C*. *turicensis* LMG 23827^T^. *In silico* analysis revealed that both plasmids encode similar groups of genes or gene clusters comprising the “backbone”of the plasmid, the same RepFIB-like origin of replication gene (*repA*), two iron acquisition systems, an aerobactin-like siderophore (named cronobactin), and an ABC ferric-iron transporter gene cluster, as well as several species-specific virulence gene determinants. It has been proposed that these plasmids may represent ^“^virulence plasmids”conferring virulence to *Cronobacter* isolates.

**Fig 2:**
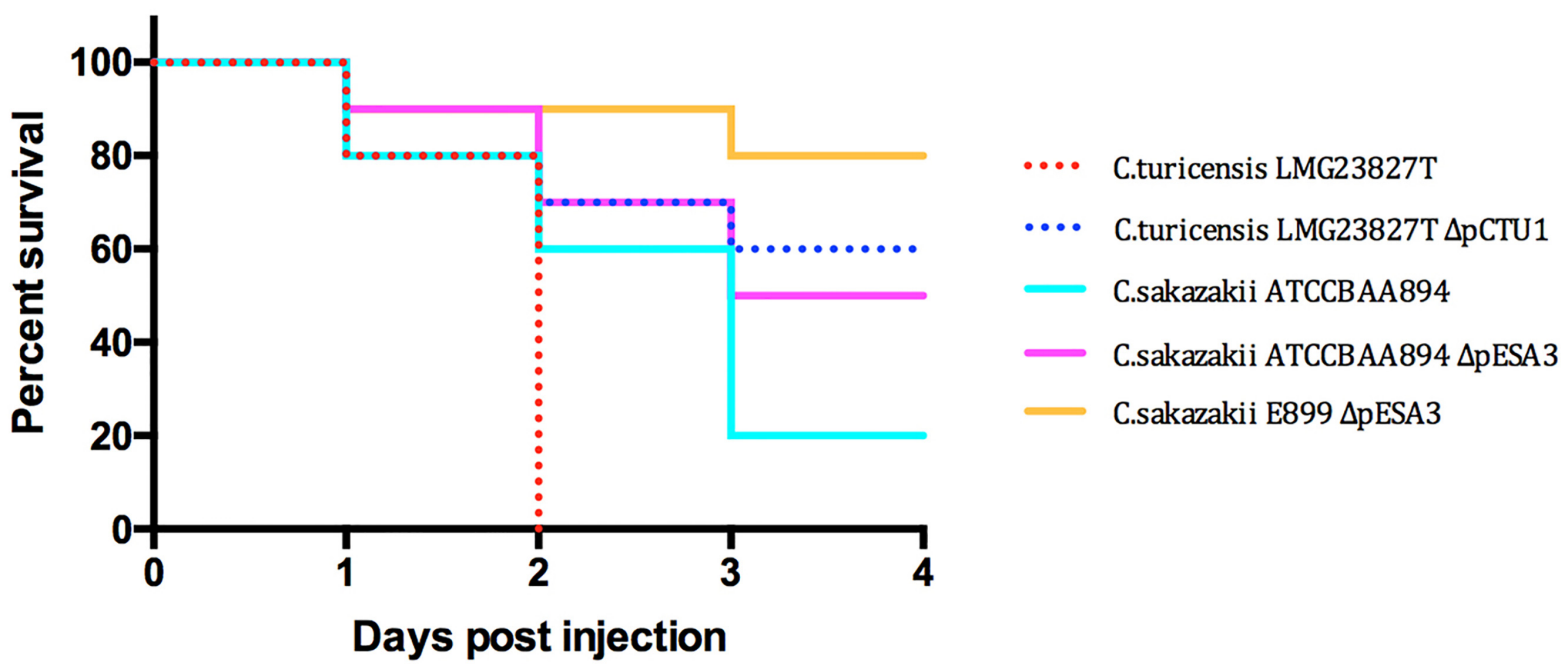
Infection experiments using *C*. *turicensis* LMG 23827^T^, *C*. *sakazakii* ATCC BAA-894 wt, the (pESA3, pCTU1) plasmid-cured strains, and *C*. *sakazakii* E899 strain naturally devoid of the pESA3 plasmid. n = 30 per strain, p = 0.0007.

In order to test the hypothesis that these RepFIB-like plasmids harbored by *Cronobacter* spp. are involved in pathogenesis, zebrafish embryo infection studies were performed using the plasmid cured strains of *C*. *turicensis* LMG 23827^T^ΔpCTU1 and *C*. *sakazakii* ATCC BAA-894ΔpESA3. As can be retrieved from Fig 2, the mortality of both plasmid-cured strains was considerably reduced compared to the results obtained with the wild type strains. In addition, when using *C*. *sakazakii* strain E899, which is naturally devoid of the pESA3 homologue in infection experiments, a maximum mortality rate of 20% was recorded at 96 hpi.

### The two putative virulence factors Cpa and Zpx play a role in *in vivo* pathogenesis

**Fig 3:**
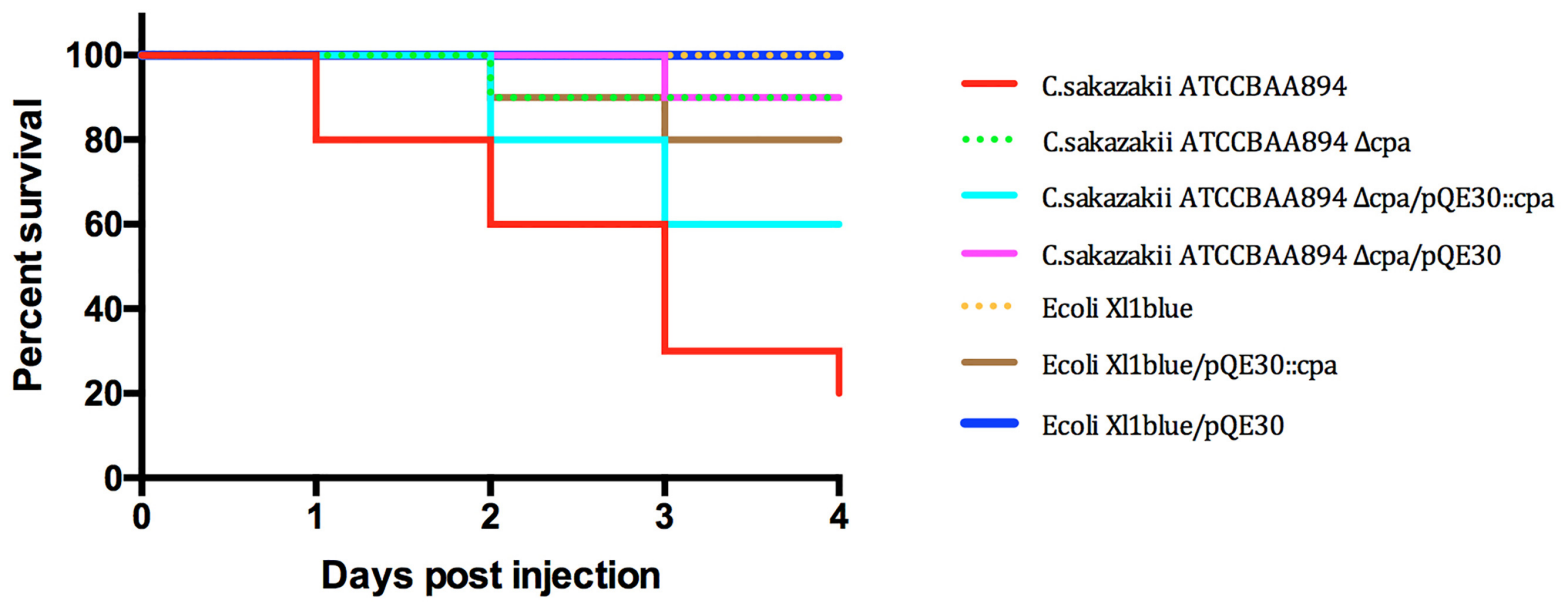
Infection experiments using the *C*. *sakazakii* ATCC BAA-894 wt, the *cpa* mutant and control strains. n = 30 embryos per strain, p < 0.0001.

In two studies by Franco et al. [25, 34], *C*. *sakazakii* plasmid pESA3 was shown to encode the putative virulence factor *Cronobacter* plasminogen activator (*cpa*) gene locus. It was demonstrated that the outer membrane protease Cpa provides serum resistance to *C*. *sakazakii* ATCC BAA-894 by proteolytically cleaving complement components, as well as by activating plasminogen and inactivating the plasmin inhibitor α2-AP. To test its influence in an *in vivo* model a knock out mutant (ATCC BAA-894*Δcpa*) was constructed and tested together with the wild type strain and ATCC BAA-894*Δcpa* complemented with the *cpa* gene in trans in the zebrafish embryo model. In addition, *E*. *coli* Xl1blue carrying the Cpa determinant in trans was included in the experiments. As shown in Fig 3, *C*. *sakazakii* ATCC BAA-894Δ*cpa* displayed only 10% mortality compared to 80% mortality observed in experiments with the wild type parental strain. Virulence was partially restored to 40% with the complemented mutant. In addition, infection experiments using *E*. *coli* Xl1blue carrying the Cpa determinant displayed a moderately elevated mortality rate (20%) when compared to *E*. *coli* wild type strain.

**Fig 4:**
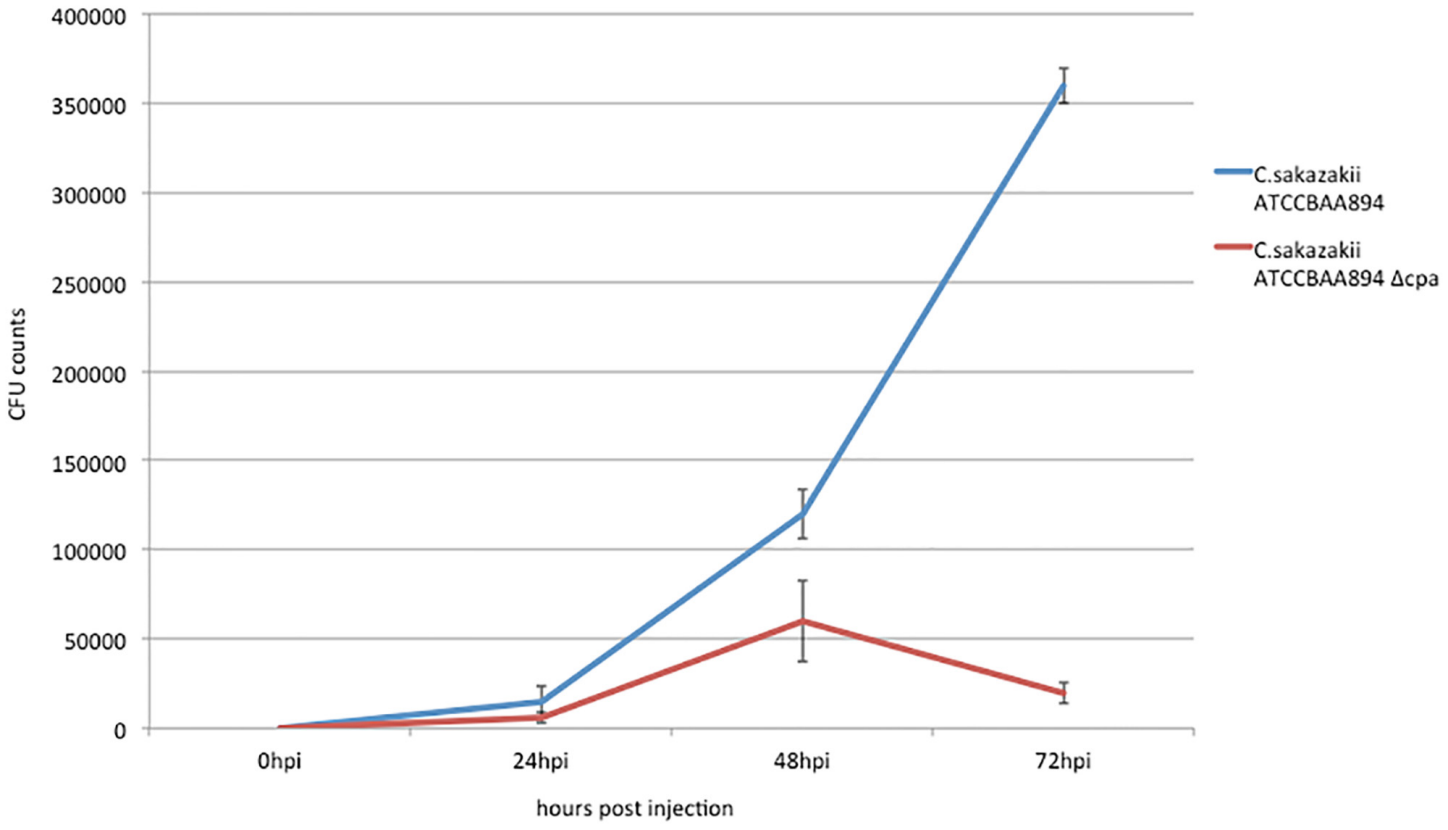
Quantitative infection experiments using *C*. *sakazakii* ATCC BAA-894 wt and the *cpa* mutant.

In order to obtain more quatitative kinetic information on the progression of the infection, growth experiments using the BAA-894 wt and *cpa* mutant (Fig 4) were carried out. Growth of the strains in the zebrafish embryo model was comparable until 24 hpi when growth slowed in the mutant and then at 48 hpi a sharp drop was observed in the bacterial counts of the mutant suggesting that the embryos were able to clear the infection. Growth curves in LB grown cultures suggested no growth defect in the mutant (S1 Fig).

**Fig 5:**
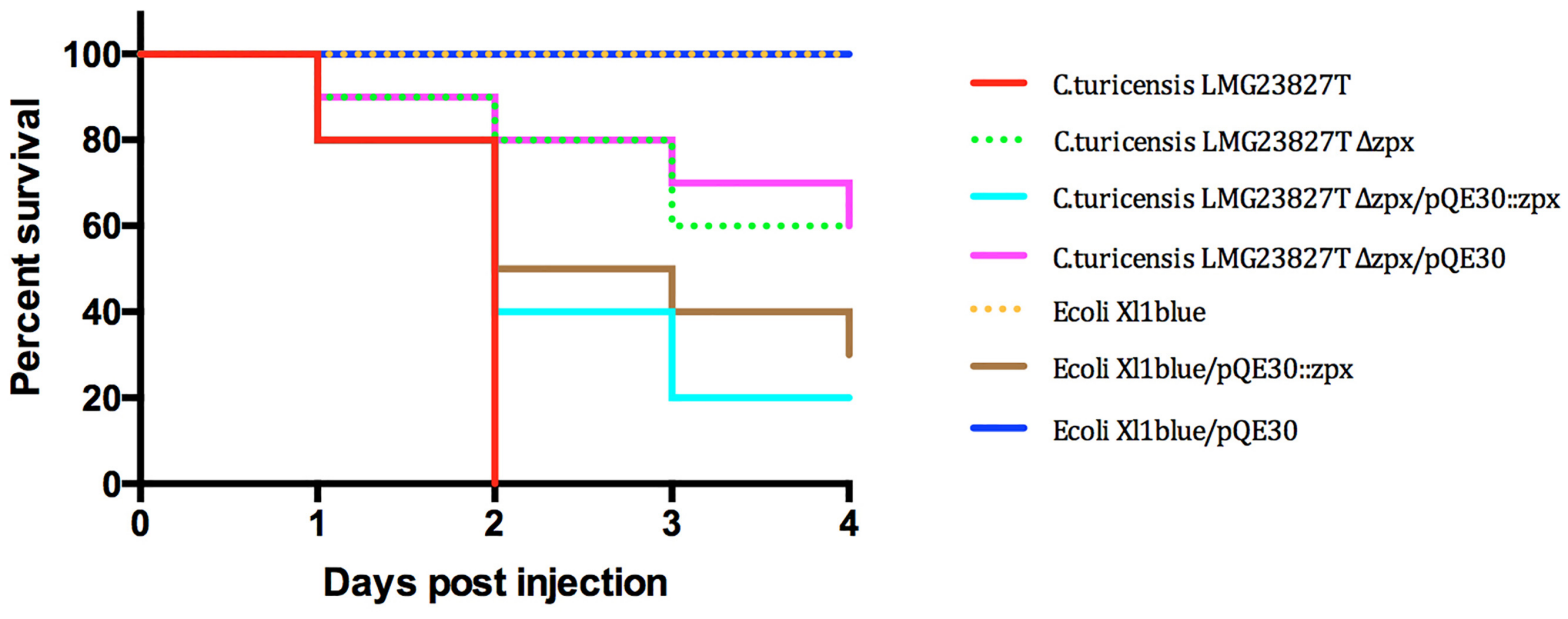
Infection experiments using the *C*. *sakazakii* ATCC BAA-894 wt, the *zpx* mutant and control strains. n = 30 embryos per strain, p < 0.0001.

Another putative virulence factor, the zinc containing metalloprotease *zpx*, was described by Kothary et al. [35]. In contrast to the above mentioned Cpa determinant, the *zpx* locus is chromosomally encoded and is supposed to be present in all *Cronobacter* species [31, 4]. Although activity of this protease against Chinese hamster ovary cells (CHO) in tissue culture has been reported, its contribution to virulence has never been tested before. A *zpx* mutant strain was constructed in *C*. *turicensis* LMG 23827^T^ and tested in the zebrafish embryo infection model together with its wild type, the complemented mutant and an *E*. *coli* Xl1blue strain carrying the Zpx determinant. The results of these experiments are depicted in Fig 5. The mortality rate in the *C*. *turicensis* LMG 23827^T^ Δ*zpx* mutant was diminished by 60% and in experiments using the complemented mutant virulence was restored to a large extent (80%). As expected, the mortality rate using the *E*. *coli* Xl1blue harbouring the *zpx* locus was also elevated to 70%, thus underpinning the role of this important viulence factor in *Cronobacter* spp. disease.

## Discussion

The results presented in this study may to some extent be correlated to the “clade theory”proposed by Grim et al. [36]. According to this hypothesis, *Cronobacter* have diverged into two clades, one consisting of *C*. *dublinensis* and *C*. *muytjensii* (Cdub-Cmuy) and the other comprised of *C*. *sakazakii*, *C*. *malonaticus*, *C*. *universalis*, and *C*. *turicensis*, (Csak-Cmal-Cuni-Ctur) from the most recent common ancestral species. It was shown, that the Cdub-Cmuy clade genomes contained several accessory genomic regions important for survival in a plant-associated environmental niche, while the Csak-Cmal-Cuni-Ctur clade genomes harbored numerous virulence-related genetic traits.

However, mortality rates such as seen with C. *dublinensis* ssp. *dublinensis* LMG 23825^T^, which is supposed to be a member of the “environmental/plant associated (Cdub-Cmuy) clade”but exhibiting an equally high mortality rate as the putative “virulence associated (Csak-Cmal-Cuni-Ctur) clade”members suggests, that additional virulence factors may exist, which are more crucial for the pathogenesis/mortality (at least in this model) than others. Whole genome comparisons are currently needed in order to elucidate other potential factors that may contribute to the highly virulent phenotype observed for this strain in our model.

One striking result was the finding that infection studies performed with the *C*. *muytjensii* ATCC 51329^T^ type strain resulted in zero mortality. It has been previously reported that this strain does not harbor a RepFIB-like “virulence plasmid”[25]. The results from our experiments suggest that the presence of the these plasmid strongly contributes to virulence. Our experiments using the plasmid-cured strains compared to parental wt strains underpinned the role of these plasmids in virulence, as mortality rates were reduced by approx. half in the plasmid-cured strains *C*. *sakazakii* BAA-894 and *C*. *turicensis* LMG 23827^T^. However, these strains did not become completely avirulent, again suggesting the presence of additional factors which contribute to pathogencity.

*In silico* analysis of pESA3 and pCTU1 revealed a high degree of similarity concerning the presence of putative virulence genes and gene clusters. However, it has also been shown, that particular virulence factors are exclusively found along species-specific evolutionary lines [25]. One of those factors—the *Cronobacter* plasminogen activator Cpa—is encoded by pESA3 and was demonstrated to be present in *C*. *sakazakii* strains [34]. This factor is supposed to play an important role in survival of the bacterium in the blood. Our results from the infection experiments using the isogenic *cpa* mutant confirmed an important role of this determinant in *in vivo* pathogenesis. From previous studies we know, that *Cronobacter* is not capable to replicate in blood [37] thus, for infection studies in zebrafish, bacteria have to be injected into the yolk saculum where they start to proliferate before traversing into the blood followed by dissemination into embryonic tissues- causing fatal bacteremia [20]. When we perfomed quantitative growth experiments in embryos using wt and *cpa* mutant we observed equal growth behaviour for both strains until 48 hpi. However, at later time points the bacterial counts decreased significantly in infections with the *cpa* mutant. We hypothesize that at time points > 48 h post infection the bacterium had already transversed into the blood where a mutant deficient of the *cpa* determinant was efficiently eliminated by the host immune system.

In conclusion, we show, that the zebrafish embryo infection model is an extremely useful tool to study the contributions of putative virulence factors whether they have been derived by *in silico* analysis or from previous *in situ* experiments. The potential of this model for use in large scale infection studies holds much promise in improving the understanding of the virulence potential of these pathogens.

## Supporting Information

S1 Fig Growth curves of the *C*. *sakazakii* ATCC BAA-894 wt and the *cpa* mutant gown in LB. Bacterial growth was monitored over 24 h at 37°C at 600 nm in 200 μl volumes of medium in 96 well plates using the Bio-Tek microplate reader (Synergy HT; Bio-Tek, Germany).
(TIF)
