# Supplementary figures and images for: Linking Genomo- and Pathotype: Exploiting the Zebrafish Embryo Model to Investigate the Divergent Virulence Potential among Cronobacter spp
Source: PLoS One. 2016 Jun 29;11(6):e0158428. doi: 10.1371/journal.pone.0158428 (PMC4927158; doi:10.1371/journal.pone.0158428)

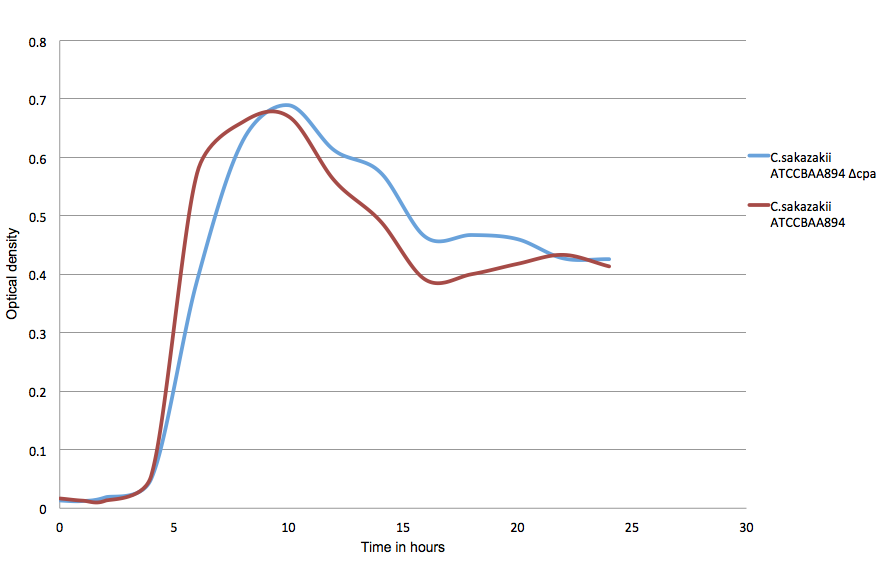

Supplement: S1 Fig — Bacterial growth was monitored over 24 h at 37°C at 600 nm in 200 μl volumes of medium in 96 well plates using the Bio-Tek microplate reader (Synergy HT; Bio-Tek, Germany). (TIF) [file pone.0158428.s001.tif]
